# Total Lipopolysaccharide from the Human Gut Microbiome Silences Toll-Like Receptor Signaling

**DOI:** 10.1128/mSystems.00046-17

**Published:** 2017-11-14

**Authors:** Eva d’Hennezel, Sahar Abubucker, Leon O. Murphy, Thomas W. Cullen

**Affiliations:** Developmental and Molecular Pathways, Novartis Institutes for Biomedical Research Inc., Cambridge, Massachusetts, USA; University of Colorado Denver

**Keywords:** microbiome, lipopolysaccharide, microbial communities, symbiosis, tolerance, Toll-like receptors

## Abstract

Cohabitation of microbial communities with the host enables the formation of a symbiotic relationship that maintains homeostasis in the gut and beyond. One prevailing model suggests that this relationship relies on the capacity of host cells and tissues to remain tolerant to the strong immune stimulation generated by the microbiota such as the activation of Toll-like receptor 4 (TLR4) pathways by lipopolysaccharide (LPS). Indeed, gut microbial LPS is thought to be one of the most potent activators of innate immune signaling and an important mediator of the microbiome’s influence on host physiology. In this study, we performed computational and experimental analyses of healthy human fecal samples to examine the TLR4 signaling capacity of the gut microbiota. These analyses revealed that an immunoinhibitory activity of LPS, conserved across the members of the order *Bacteroidales* and derived from an underacylated structural feature, silences TLR4 signaling for the entire consortium of organisms inhabiting the human gut. Comparative analysis of metagenomic data from the Human Microbiome Project and healthy-donor samples indicates that immune silencing via LPS is a microbe-intrinsic feature in all healthy adults. These findings challenge the current belief that robust TLR4 signaling is a feature of the microbiome and demonstrate that microbiome-derived LPS has the ability to facilitate host tolerance of gut microbes. These findings have broad implications for how we model host-microbe interactions and for our understanding of microbiome-linked disease.

**IMPORTANCE** While the ability for humans to host a complex microbial ecosystem is an essential property of life, the mechanisms allowing for immune tolerance of such a large microbial load are not completely understood and are currently the focus of intense research. This study shows that an important proinflammatory pathway that is commonly triggered by pathogenic bacteria upon interaction with the host is, in fact, actively repressed by the bacteria of the gut microbiome, supporting the idea that beneficial microbes themselves contribute to the immune tolerance in support of homeostasis. These findings are important for two reasons. First, many currently assume that proinflammatory signaling by lipopolysaccharide is a fundamental feature of the gut flora. This assumption influences greatly how host-microbiome interactions are theoretically modeled but also how they are experimentally studied, by using robust TLR signaling conditions to simulate commensals. Second, elucidation of the mechanisms that support host-microbe tolerance is key to the development of therapeutics for both intestinal and systemic inflammatory disorders.

## Abstract (precis)

While the ability for humans to host a complex microbial ecosystem is an essential property of life, the mechanisms allowing for immune tolerance of such a large microbial load are not completely understood and are currently the focus of intense research. This study shows that an important proinflammatory pathway that is commonly triggered by pathogenic bacteria upon interaction with the host is, in fact, actively repressed by the bacteria of the gut microbiome, supporting the idea that beneficial microbes themselves contribute to the immune tolerance in support of homeostasis. These findings are important for two reasons. First, many currently assume that proinflammatory signaling by lipopolysaccharide is a fundamental feature of the gut flora. This assumption influences greatly how host-microbiome interactions are theoretically modeled but also how they are experimentally studied, by using robust TLR signaling conditions to simulate commensals. Second, elucidation of the mechanisms that support host-microbe tolerance is key to the development of therapeutics for both intestinal and systemic inflammatory disorders.

## INTRODUCTION

The human gut microbiome comprises a large and dynamic population of microorganisms representing one of the most densely populated ecosystems known (1). This microbial consortium provides many benefits to its host, including key signals that shape gastrointestinal development, immune maturation, vitamin production, extraction of otherwise indigestible carbohydrates from the diet, and pathogen resistance (2). However, this presents the challenge for the host of containing, and remaining immunologically tolerant to, a microbial load in excess of 10^12^ cells/ml (1). Elaborate mechanisms are required to modulate and preserve this symbiotic relationship. To date, mechanisms reported to maintain this symbiosis rely on host-driven tolerance, including the physical barriers of the oriented epithelium and mucosa, secretion of antimicrobial peptides and secreted antibodies, or negative feedback loops in NF-κB signaling (3). Several mechanisms are also at play that contain both the risk of infection and the amplitude of the immune response (4). In recent years, studies have reported examples of microorganisms themselves promoting the expansion of Foxp3^+^ regulatory T cells (5, 6), the suppression of tumor necrosis factor alpha (TNF-α) production (7, 8), and the maintenance of the gut epithelium (9), suggesting that the microbiota could play a role in shaping host immune responses. However, specific microbiota-derived molecular mediators of host tolerance are still largely unknown.

We recently reported that the commensal organism *Bacteroides dorei* produces an antagonistic form of lipopolysaccharide (LPS) that can influence the susceptibility of children to allergies and autoimmunity (10). While the ability of some LPS isoforms to inhibit Toll-like receptor 4 (TLR4) signaling has been reported (11), their broader implications with regard to gut health and disease remain unexamined. Here we directly extracted the total LPS from fecal samples from healthy adult humans and found that the LPS produced by the consortium of gut-resident microbes potently antagonizes the host TLR4 pathway. Using metagenomic sequencing, we further delineated strain level contributions to the gut LPS pool and found that numerous other members of the order *Bacteroidales*, which are the dominant Gram-negative bacteria in the healthy human gut microbiome (12), produce antagonistic forms of LPS, thus driving immune silencing for the entire microbial community. These findings undermine the current accepted paradigm that gut microbial communities possess a robust TLR4 signaling capacity against which the immune system needs to be heavily tolerized (13). Ours is the first report describing a phylum-wide microbiome-intrinsic mechanism actively damping innate immune activation in the healthy gut and redefines how we envision the immunological dynamics of the host-microbiota relationship.

## RESULTS

### Total LPS produced in the adult human gut is immunoinhibitory.

**FIG 1:**
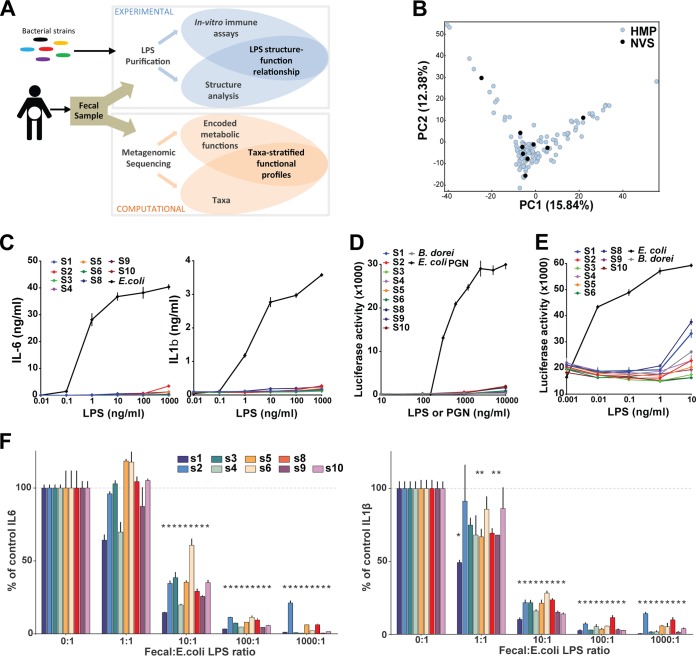
Total LPS from the human gut microbiome is immunoinhibitory. (A) Schematic diagram of sample processing and analysis in this study. (B) Principal-component analysis plot comparing the HMP1 sample set (blue) and a new set of nine healthy-donor (Novartis [NVS]) samples (black). (C) Human PBMCs were stimulated in the presence of increasing amounts of fecal LPS. IL-6 and IL-1β concentrations in supernatants were measured after 20 h of culture. *E. coli* LPS was included as a control. (D and E) HEK-293 cells expressing an NF-κB–luciferase reporter and either hTLR2 and hCD14 (D) or hTLR4, hMD2, and hCD14 (E) were stimulated in the presence of increasing doses of fecal LPS from each donor, *B. dorei* LPS, or *E. coli* PGN. A sterile-water negative control was included in each experiment (not shown). Luciferase activity was measured after 6 h of activation. Data shown are the mean ± the standard deviation of triplicate evaluations from one out of three independent experiments. (F) PBMCs were cotreated with 1 ng/ml *E. coli* LPS and increasing doses of fecal LPS from the donors indicated. IL-6 and IL-1β concentrations in supernatants were measured after 20 h of culture and compared to those obtained with LPS treatment alone (0:1). Data shown are the mean ± the standard deviation of triplicates in one representative experiment. *, corrected *P* value of <0.05.

Stool samples were collected from nine healthy adults and analyzed as illustrated in Fig. 1A. The microbial composition of each sample was analyzed by metagenomic whole-genome sequencing (WGS), which revealed an overall bacterial composition that was similar to the general microbial landscape depicted in Human Microbiome Project 1 (HMP1) (14) (Fig. 1B), suggesting that these samples are representative of the general population.

LPS was purified from each donor as previously described (15) and further processed as previously described by Hirschfeld et al. to remove contaminating lipoproteins and yield purified LPS (16). The immunostimulatory potency of each sample of fecal LPS was then assessed by stimulating human primary PBMCs. The production of six inflammatory cytokines, interleukin-10 (IL-10), IL-6, IL-8, TNF-α, IL-12p70, and IL-1b, was measured in parallel. All cytokines that reached detectable levels (IL-10, IL-6, IL-8, IL-1b, and TNF-α) showed similar patterns of production, in accordance with their common regulation by NF-κB activation downstream of LPS. IL-6 and IL-1b are shown as representative results. All LPS samples showed ≥2 orders of magnitude lower stimulatory potency than purified *Escherichia coli* LPS (no. 1, 2, 5, 8, and 10), and many (no. 3, 4, 6, and 9) were immunologically silent up to the largest dose tested on primary peripheral blood mononuclear cells (PBMCs; 1 mg/ml) (Fig. 1C). Similar results were obtained when stimulating human TLR2 (hTLR2) and hTLR4--NF-κB reporter cell lines, confirming the absence of direct signaling through these LPS-sensing signaling pathways (Fig. 1D and E). Hence, total fecal LPS has a very limited capacity to activate the TLR4--NF-κB pathway and elicit the production of inflammatory cytokines. This result is particularly striking given that the gut microbiome is thought to elicit robust TLR4 signals and that total fecal LPS is likely derived from diverse microbial origins, including species known to produce potent forms of LPS (e.g., gammaproteobacteria). We further examined the immunomodulating properties of fecal LPS by cotreating PBMCs with fecal LPS prior to stimulation with *E. coli* LPS to assess their ability to interfere with immune stimulation. Fecal LPS from each donor potently inhibited the cytokine (IL-6, IL-1b) production elicited by *E. coli* LPS. Most fecal samples reached significant inhibition at a 1:10 *E. coli*-to-fecal LPS ratio, while some reached significant IL-1b inhibition at a 1:1 ratio (Fig. 1F). Hence, total fecal LPS is a potent inhibitor of TLR4 stimulation.

### Gut LPS derives primarily from *Bacteroidales.*

**FIG 2:**
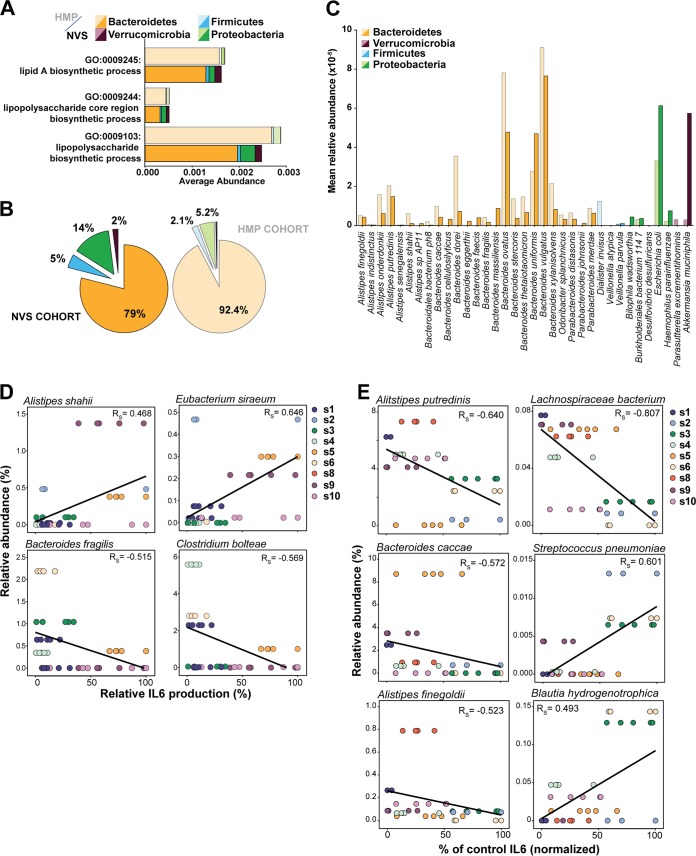
*Bacteroidetes* bacteria are the main contributors to LPS biosynthesis in the human gut microbiome. (A) Average abundance per sample of genes related to the three main LPS biosynthesis-related functions. (B) Relative contributions of the different phyla to the total LPS-encoding capacity of the gut microbiome determined in both cohorts. The three functions of LPS biosynthesis were pooled. The darker-color set is HMP samples, and the lighter-color set is NVS samples. (C) Contribution of individual species to LPS biosynthesis functions. The average relative abundances of genes related to any of the three LPS-related GO functions are shown for individual species within each phylum. Only species detected in >50% of the donors in each data set are shown. The darker-color set is HMP samples, and the lighter-color set is NVS samples. (D) Correlation of individual-species abundance with stimulation of IL-6 production as determined in panel A. (E) Correlation of individual-species abundance with inhibition of IL-6 production as determined in panel B. Each data point represents one independent experiment. Rs, Spearman rho.

Previous studies have demonstrated that a few bacterial species known to be present in the human gut microbiome produce nonstimulatory or immunoinhibitory LPS (10, 17), which prompted us to examine which bacterial species were present in these samples that contributed to the total pool of fecal LPS. Metagenomic WGS of the fecal samples revealed that the distribution of the main phyla, as determined by using MetaPhlAn2 (18), was similar in the nine volunteers and not distinguishable from the 140 healthy-donor samples in HMP1 (Fig. 1B; see Fig. S1A in the supplemental material), suggesting that these samples are representative of the general population. We also analyzed both sample cohorts by using the HUMAnN2 algorithm (unpublished; http://huttenhower.sph.harvard.edu/HUMAnN2) to determine encoded functional pathways and inferred gene ontology (GO) functions with UniRef50 gene families. As previously described for the HMP1 cohort, the distribution of the encoded functions is similar across all samples and was also comparable within the newly collected samples (Fig. S1B). To identify which strains are contributing to LPS production in the human gut, we focused on the GO terms relevant to LPS biosynthesis (Fig. 2A and B). Both in fresh samples and in the HMP1 data set, *Bacteroidetes* species dominate the three main GO terms related to LPS biosynthesis. Overall, *Bacteroidetes* species contribute 79% of the LPS biosynthesis in healthy volunteers and 92.4% of that in HMP1 samples (Fig. 2B). In contrast, proteobacteria are minor contributors to LPS biosynthesis, with an average of 14% of the total produced LPS being of *E. coli* origin in volunteers; in HMP1 donors, the percentage is 5.2% (Fig. 2B; Fig. S2C). The resulting estimated average *Bacteroidetes*-to-*E. coli* LPS ratio in the gut would be between 6:1 in volunteers and 18:1 in the HMP1 cohort. The abundances of the *Bacteroidales* species, and therefore their likely contribution to the LPS pool, are shown in Fig. 2C. *Bacteroides* species contributing to LPS are diverse but follow similar trends. Notably, *Bacteroides ovatus*, *B. uniformis*, and *B. vulgatus* dominate LPS production in both cohorts (Fig. 2C).

10.1128/mSystems.00046-17.1 FIG S1  (A) Distribution of species in individual samples into the five main phyla in the new-sample (S1 to S10) and HMP1 sample sets. (B) Distribution of encoded functional pathways in individual samples of the new data set (NVS samples) or the HMP1 data set (HMP). (C) Contribution of the phylum *Bacteroidetes* to the total LPS-encoding capacity of the gut microbiome relative to that of other phyla. Data are presented for each individual donor. The darker-color set is HMP samples, and the lighter-color set is NVS samples. Download FIG S1, TIF file, 0.8 MB. Copyright © 2017 d’Hennezel et al. 2017 d’Hennezel et al. This content is distributed under the terms of the Creative Commons Attribution 4.0 International license.

10.1128/mSystems.00046-17.2 FIG S2  Stimulation of PBMCs by *Bacteroidales* LPS. (A and B) Human PBMCs were stimulated in the presence of increasing amounts of LPS from the *Bacteroidales* species indicated. TNF-α and IL-1β concentrations in supernatants were measured after 20 h of culture. *E. coli* LPS was included as a control. Data shown are the mean ± the standard deviation of triplicates in one representative experiment. (C and D) LPS isolated from the species indicated. Human PBMCs were cotreated with zymosan and increasing doses of *Bacteroidales* LPS. TNF-α and IL-1β concentrations in supernatants were measured after 20 h of culture. Treatment with zymosan alone was included as a control (black bar). Data shown are the mean ± the standard deviation of triplicates in one representative experiment. Download FIG S2, TIF file, 0.6 MB. Copyright © 2017 d’Hennezel et al. 2017 d’Hennezel et al. This content is distributed under the terms of the Creative Commons Attribution 4.0 International license.

We next examined whether the bacterial composition of the samples would correlate with the potency of stimulation or inhibition in these samples (Fig. 2D and E). We could not identify a strong correlation between function and composition at the phylum level and could only identify weak-to-moderate correlations between a few individual species and the stimulatory potency of individual fecal LPS samples (Fig. 2D). However, we found that the abundance of several *Bacteroidales* species (*Alistipes putredinis*, *Bacteroides caccae*, *Alistipes finegoldii*) show a moderate-to-strong correlation with the inhibition of IL-6 cytokine production (Fig. 2E), as well as TNF-α and IL-1β production (data not shown). Cytokine production and trends were similar for all of the cytokines measured. Interestingly, we also identified several Gram-positive species whose abundance correlates with functionality, notably, *Lachnospiraceae* bacterium 5_1_63FAA (Fig. 2E). However, these strains are present at very low abundance (<0.5% of the genomic pool), Gram-positive strains do not contribute to the LPS pool, and it remains unclear what indirect impact they may have on LPS signaling. Our results show that *Bacteroidetes* species are by far the most abundant contributors to LPS biosynthesis functions in the healthy human intestinal microbiota, consistent with their high abundance relative to other Gram-negative species in the gut (12, 14).

### Immunosuppressive LPS in *Bacteroidales* species*.*

**FIG 3:**
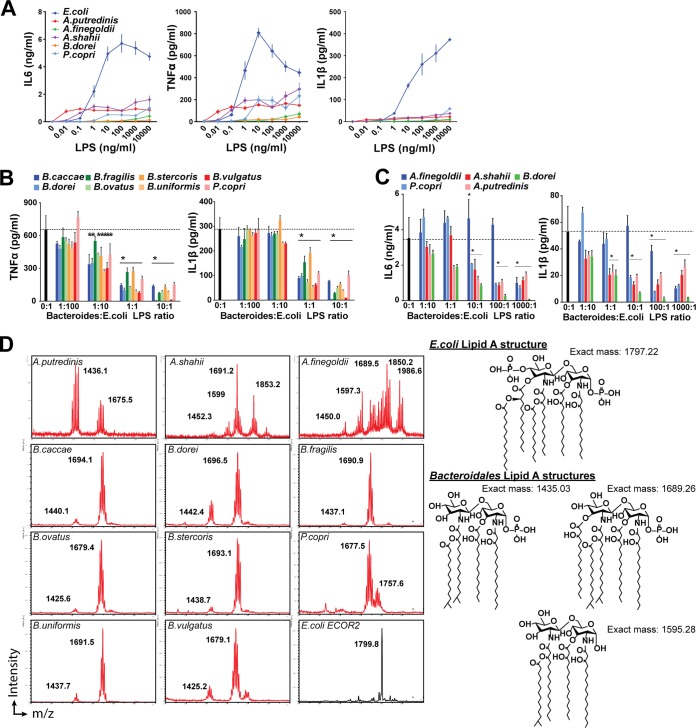
*Bacteroidales* LPS inhibits TLR stimulation. (A to C) LPS was isolated from the species indicated. (A) Human PBMCs were stimulated in the presence of increasing amounts of LPS from the *Bacteroidales* species indicated. IL-6, TNF-α, and IL-1β concentrations in supernatants were measured after 20 h of culture. *E. coli* LPS was included as a control. Data shown are the mean ± the standard deviation of triplicates in one representative experiment. (B and C) Human PBMCs were cotreated with *E. coli* LPS and increasing doses of LPS from *Bacteroidales* (B) and *Alistipes* (C) spp. TNF-α and IL-1β concentrations in supernatants were measured after 20 h of culture. *E. coli* LPS is shown as a control (dotted line). Data shown are the mean + the standard deviation of triplicate measurements in one representative of three or more independent experiments. *, corrected *P* value of <0.05. (C) MALDI-TOF MS analysis of the lipid A moiety of individual *Bacteroidales* and *E. coli* LPSs. *m/z* values are indicated for major peaks. On the right are the lipid A structures associated with the main *m/z* values.

We had previously shown that LPS from several members of the order *Bacteroidales* were unable to activate TLR4, consistent with literature reports (10, 17, 19), and that *B. dorei* produces an immunoinhibitory form of LPS (10). Thus, we examined whether immunosilent LPS is produced not only by *Bacteroides* and *Prevotella* spp*.* but also by additional gut-associated *Bacteroidales* bacteria (i.e., *Alistipes* spp*.*) not examined in our previous studies and whether immunoinhibitory LPS is a common, phylum-wide feature of the order *Bacteroidales*. We isolated LPS from 11 *Bacteroidales* strains and tested its capacity to elicit inflammatory responses from primary human PBMCs. All three *Alistipes* spp. tested produced an LPS with low immunostimulatory capacity, similar to other members of the order *Bacteroidales* (Fig. 3A; Fig. S2A and B). We then tested its capacity to modulate the response of primary human PBMCs to stimulation by *E. coli* LPS. Across the order *Bacteroidales*, LPS induces a potent reduction of TNF-α or IL-6 production. Production and trends were similar for all of the cytokines measured. This included a significant suppression at a 1:10 *Bacteroidales-*to*-E. coli* LPS ratio for all of the species tested, with the notable exception of *Alistipes finegoldii*, which only suppresses at higher ratios (100:1 or 1,000:1) (Fig. 3B and C). Comparable suppression was seen with IL-1β (Fig. 3B and C), and unexpectedly, similar results were obtained for inhibition of zymosan-elicited cytokine production (Fig. S2C).

Inhibitory forms of LPS have been previously described whose activity is derived from variations in the structure of the lipid A moiety (20, 21). The lipid A domain of LPS is responsible for the endotoxic properties associated with LPS due to recognition by the TLR4 complex and downstream activation of the NF-κB pathway (22). A reduction in the number of lipid A acyl chains by bacteria has been shown to modulate the recognition of LPS by TLR4 (23). Structural analysis of the lipid A domain from all of the members of the order *Bacteroidales* studied by matrix-assisted laser desorption ionization--time of flight mass spectrometry (MALDI-TOF MS) analysis revealed masses with a mass/charge ratio (*m*/*z*) of <1,700, consistent with the presence of underacylated lipid A structures (Fig. 3D). Specifically, all *Bacteroides* spp*.* and *Prevotella copri* showed a dominant peak having an *m*/*z* of 1,677.5 to 1,696.5, consistent with the [M − H]^−^ ions of a penta-acylated form of lipid A. Also common but with variable abundance is a peak at an *m*/*z* of approximately 1,435, consistent with the [M − H]^−^ ions of a tetra-acylated form of lipid A. Variation in determined mass is consistent with variable length in acyl chains. The spectrum of *P. copri* shows an additional minor peak with an *m*/*z* of 1,757.6, consistent with the addition of a phosphate group but otherwise identical to other members of the order *Bacteroidales*. All *Alistipes* spp. showed masses consistent with underacylated lipid A structures in addition to larger masses in *A. shahii* and *A. finegoldii* of unknown significance (Fig. 3D). Our analysis indicate that immunosilent and immunoinhibitory underacylated lipid A structures are conserved across the order *Bacteroidales*.

In a parallel effort to identify novel microbe-derived immunomodulatory molecules, we screened extracellular polymeric substances (EPS), a secreted cell-associated matrix often containing biologically active molecules (24). Given the intimate association of the commensal gut microbes with mucosal surfaces, EPS represent an unexplored niche for immunologically active factors. We extracted the EPS of 29 bacterial strains selected to comprise representatives of the four main phyla of the human gut microbiome (7) (Fig. S3A). We then assessed the capacity of these EPS extracts to modulate inflammation in primary human immune cells and showed that TLR2 (zymosan)- and TLR4 (LPS)-driven cytokine production is reduced 4- to 20-fold by extracts from several strains, mainly members of the order *Bacteroidales* (Fig. S3B to E). Using an in-gel LPS staining procedure (25), we determined that the EPS extracts from *Bacteroides* strains exhibit high LPS content (Fig. S3F). These results further confirm that LPS from members of the order *Bacteroidales* potently antagonizes TLR signaling and suggest that the EPS matrices of commensal organisms are a likely vehicle for immunomodulatory factors.

10.1128/mSystems.00046-17.3 FIG S3  EPS extracts from bacterial species of the human microbiome have immunomodulatory properties. (A) Phylogenetic tree of the 120 most abundant bacterial strains in the human gut microbiome. EPS extracts were prepared from 29 strains (red). (B and C) Human monocyte-derived dendritic cells (B and C) or PBMCs (D and E) were cotreated with individual EPS extracts and zymosan (B and D) or LPS (C and E). The TNF-α concentration in supernatants was compared to that obtained with zymosan or LPS treatment alone. Data shown are the mean of triplicate measurements in one representative of three or more independent experiments. (F) EPS extracts from Gram-negative species were analyzed by SDS-PAGE, followed by Pro-Q Emerald staining for glycoproteins. Purified *E. coli* LPS was loaded as a control. Download FIG S3, TIF file, 2.2 MB. Copyright © 2017 d’Hennezel et al. 2017 d’Hennezel et al. This content is distributed under the terms of the Creative Commons Attribution 4.0 International license.

## DISCUSSION

Immune mechanisms underlying intestinal commensalism have yet to be fully elucidated. Since the realization that our intestinal tract is host to billions of bacteria in the absence of an overwhelming immune response, the mechanisms that maintain this relationship have been the subject of intense study. Here we formally demonstrate that the total LPS produced by the human gut microbiome not only is itself nonimmunogenic but also inhibits TLR4-dependent cytokine production (Fig. 1B to F). We further show that production of immunoinhibitory forms of LPS is a common feature across members of the order *Bacteroidales* (Fig. 3), which are the major contributors of LPS synthesis in the human gut (Fig. 2). Previous publications have demonstrated that distinct structural features of the lipid A domain, produced by a few bacterial species, interfere with proper TLR4-MD2 signaling via competitive inhibition (17, 19). The exact mechanism of signaling inhibition by feces-derived LPS has not been demonstrated in this study but is likely to be identical to previously described mechanisms.

We had previously reported on the immunoinhibitory function of LPS produced by *B. dorei* and the role it likely plays in precluding proper immune education in infants genetically predisposed to type 1 diabetes, thus favoring the development of allergies and autoimmunity (10). We also observed a general overabundance of *Bacteroidetes* bacteria in these infants, which, in light of the findings reported here, suggests that the collective contribution of all *Bacteroides* spp. likely enhanced the disease susceptibility of these children. Any effort to target the gut microbial community in these infants for therapeutic purposes should therefore likely focus broadly on all *Bacteroidetes* bacteria, not just *B. dorei*.

Our findings also shed new light on a number of discoveries made in recent years that suggest a link between the inflammatory stimulation arising from the intestinal lumen and local or peripheral inflammatory disorders. Inflammatory bowel disease (IBD) has been associated with a bloom of *Proteobacteria* (26, 27). Interestingly, treatment with the aminoglycoside antibiotic gentamicin reduces the abundance of *Proteobacteria* and results in a dominance of the gut flora by *Bacteroidetes*, leading to protection from colitis in a murine model of IBD. Conversely, vancomycin treatment has been shown to favor an increase in *Proteobacteria* and does not prevent disease (28, 29). While the specific role of inflammatory LPS in the etiology and recovery of IBD remains to be elucidated, it is possible that differences in LPS immunogenicity between *Bacteroidetes* and *Proteobacteria* underlie these observations. In obesity, a common hypothesis is that intestinal LPS leaks out into the circulation, leading to subclinical, chronic inflammation in peripheral adipose tissues, altering their metabolic functions (30–32). However, our findings suggest that freely circulating LPS coming from the gut lumen tends to prevent, rather than favor, inflammation. Interestingly, obesity is associated with a decrease in *Bacteroidetes* species, relative to an increase in *Firmicutes* species, which are mostly Gram-positive bacteria (33). Thus, the intestinal LPS composition in these patients could be shifted away from anti-inflammatory *Bacteroidetes* LPS subtypes in favor of inflammatory LPS subtypes, possibly producing a more inflammatory LPS. Finally, the current methodologies used to quantify peripheral exposure to intestinal LPS are also sensitive to hypoacylated LPS structures; therefore, no assay currently exists that can distinguish inhibitory from inflammatory LPS in the periphery. While we do not dispute the effect of inflammatory LPS on the metabolic profile of peripheral tissue, our findings warrant caution in interpreting the significance of peripheral LPS levels in studies attempting to connect microbiome LPS and peripheral inflammation.

Most importantly, our findings challenge the current perception of the mechanisms regulating the cohabitation of the gut microbiota and the host. Commensalism is commonly thought of as an equilibrium of two powerful forces, a heavy bacterial load endowed with high inflammatory potential and a well-protected host with a tightly regulated immune system. This model has long guided efforts to elucidate the mechanisms underlying commensalism between the host and the gut microbiome. Experimentally, this has translated into the use of potent inflammatory LPS from pathogenic organisms to simulate the interaction with the microbiome *in vitro* and *in vivo* (34). In contrast, our findings show that total gut microbiome LPS is, in fact, overall immunoinhibitory. However, other bacterial factors contribute to the immunogenicity of the microbiome. Notably the production of the TLR2 ligand peptidoglycan (PGN) is ubiquitous in intestinal bacteria. While it has been shown that some pathogenic bacteria can use autolytic enzymes to alter their PGN to reduce the stimulation of TLR2, whether commensal bacteria produce altered forms of PGN or bear attenuated immune functions remains to be determined (35, 36). The inhibition of zymosan-mediated TLR2 stimulation by *Bacteroides* EPS and LPS we have observed also points to the possibility of a more widespread inhibition of immunogenicity by *Bacteroidales* that would extend to other signaling pathways beyond TLR4.

In recent years, a few reports have described contributions of individual species to specific mechanisms interfering with NF-κB signaling or actively modulating the TLR responsiveness in the gut epithelium (37, 38). Importantly, the present report is the first describing a phylum-wide, microbiome-derived mechanism that actively promotes immune tolerance of gut microbiota. Until now, the innate immune inflammatory potential of the commensal microbiota has likely been overestimated and the signaling capacity of the gut microbiome must be reassessed to accurately model the impact that resident commensal microbes have on health and disease.

## MATERIALS AND METHODS

### Bacterial strains and growth conditions.

The bacterial strains used in this study are summarized in tables at https://figshare.com/s/5e56cc1a347ef4f1de49. All strains were started from 20% glycerol stocks stored at −80°C, plated onto brain heart infusion (BHI) agar supplemented with hemin and vitamin K (Teknova; B1093), and grown anaerobically at 37°C. Liquid cultures of all strains were started from a single colony inoculated into 1,000 ml of BHI liquid medium supplemented with 10 ml of vitamin K-hemin solution (BD; 212354), 10 ml of trace minerals (ATCC; MD-TMS), 10 ml of trace vitamins (ATCC; MD-VS), and 50 ml of fetal bovine serum (HyClone; SH30071) and grown anaerobically for at least 48 h at 37°C. A flexible anaerobic chamber (Coy Laboratory Products) containing 20% CO_2_, 10% H_2_, and 70% N_2_ was used for all anaerobic microbiology steps.

### EPS extracts.

EPS matrix was extracted from all strains as previously described (7). Briefly, 250-ml volumes of 24-h cultures were recovered by centrifugation at 18,400 × *g* for 10 min (4°C). The prewashed cell pellet was suspended in 8 ml of phosphate-buffered saline by vortexing for 5 min, allowing the cell-bound EPS to dissolve. Planktonic cells were subsequently pelleted by centrifugation at 18,400 × *g* for 10 min (4°C). The supernatant was then carefully removed, filter sterilized with a 0.2-µm-pore-size filter, and added to 4 volumes of ice-cold absolute ethanol to precipitate the EPS. After centrifugation at 3,300 × *g* for 30 min, the precipitated-EPS pellet was washed with 70% ethanol, lyophilized, and then stored at −20°C. For further experiments, lyophilized EPS fractions were normalized by being dissolved in ultrapure water at the desired concentration. An average of 5 mg of lyophilized extract was recovered for each strain by using this protocol.

### Human cell isolation and differentiation for immune stimulation assays.

Blood buffy coats were obtained from healthy volunteers after informed consent was obtained. The study protocol and any amendments were reviewed and approved by an independent review board (New England IRB, Newton, MA) before the start of the study. This study was conducted in accordance with the ethical principles of the Declaration of Helsinki.

PBMCs were freshly isolated from blood by Ficoll-Hypaque gradient centrifugation as previously described (39). Monocyte-derived dendritic cells were differentiated *in vitro* from freshly isolated human monocytes as previously described (39). Briefly, CD14^+^ monocytes were isolated from freshly purified PBMCs by negative selection and magnetic bead sorting (Miltenyi). Cells were then incubated in complete RPMI 1640 in the presence of 50 ng/ml recombinant human granulocyte-macrophage colony-stimulating factor and 20 ng/ml recombinant human IL-4 (R&D Systems) for 7 days. For all experiments using human donor cells, data were generated independently with at least two donors. A representative data set was selected for incorporation into the figure.

### LPS purification and analysis.

To isolate the total LPS from a fecal sample, approximately 5 g of fecal material was homogenized into 10 ml of endotoxin-free water with a gentleMACS Dissociator. The resulting fecal slurry was allowed to settle for 5 min, allowing large particles to settle, and the supernatant was lyophilized for LPS purification. LPS purification from fecal material was performed with 500 mg of lyophilized material but otherwise performed as described for bacterial strains below.

For LPS isolation from bacterial strains, LPS from all strains was isolated from a 1,000-ml liquid culture grown under standard conditions for ~48 h by the hot water-phenol method as previously described (15). To remove trace amounts of endotoxin protein, phenol-purified LPS was further treated as previously described (16), with the modifications described below. Following the final ethanol precipitation, LPS was lyophilized to determine the yield with a Mettler Toledo XS105 Dual Range analytical balance (sensitivity, ≥0.1 ng) and resuspended in HyPure cell culture grade endotoxin-free water (HyClone) to a final concentration of 1 mg/ml without the addition of triethanolamine. To confirm the purity and normalization of feces-derived LPS, the final product was visualized with the Pro-Q Emerald 488 in-gel staining kit (Thermo Fisher Scientific) in accordance with the manufacturer’s instructions. In all cases, the Pro-Q Emerald 488 in-gel staining kit indicated a purity identical to that of LPS purified from bacterial isolates. However, given the complex molecular nature of human fecal material, our analysis does not exclude the possibility of contaminating substances in feces-derived LPS and it is not considered ultrapure.

### *In vitro* LPS stimulation assays and competition assays.

PBMCs (10^5^) or monocyte-derived dendritic cells (5 × 10^4^) were incubated in the presence of LPS purified from the bacterial isolates indicated at doses ranging from 10^−3^ to 10^4^ ng/ml for 18 to 20 h. For inhibition assays, cells were plated in medium. LPS purified from the strain indicated was then added, followed immediately by 100 pg/ml LPS purified from *E. coli*. Supernatants were collected after 18 to 20 h of culture and analyzed with the cytokine bead array human inflammation kit (BD Biosciences) in accordance with the manufacturer’s instructions. This kit analyzes the levels of IL-10, IL-6, IL-8, TNF-α, IL-12p70, and IL-1b in the same samples. Groups were compared by using a two-tailed nonhomoscedastic *t* test corrected for multiple testing by the Sidak-Bonferroni method with GraphPad Prism software.

### Stool sample collection and DNA extraction.

Stool samples were collected from healthy volunteers after informed consent was obtained. The study protocol and any amendments were reviewed and approved by an independent review board (Western IRB, Puyallup, WA) before the start of the study. Stool samples were collected by the participants in the morning and transported to the Novartis Institute for Biomedical Research in Cambridge, MA, on the same day. Samples were then stored at −80°C until shipping to the Broad Institute for DNA extraction. DNA extractions from stool samples were carried out with the QIAamp DNA Stool minikit (QIAGEN).

### Metagenome library construction.

Metagenomic whole-genome shotgun sequencing libraries were prepared as follows. Metagenomic DNA samples were quantified by Quant-iT PicoGreen dsDNA Assay (Life Technologies, Inc.) and normalized to a concentration of 50 pg/μl. Illumina sequencing libraries were prepared from 100 to 250 pg of DNA with the Nextera XT DNA Library Preparation kit (Illumina) in accordance with the manufacturer’s recommended protocol, with reaction volumes scaled accordingly. Batches of 24, 48, or 96 libraries were pooled by transferring equal volumes of each library with a Labcyte Echo 550 liquid handler. Insert sizes and concentrations for each pooled library were determined with an Agilent Bioanalyzer DNA 1000 kit (Agilent Technologies).

### Sequencing and analysis of metagenomic samples.

Metagenomic whole-genome shotgun sequencing was performed essentially as previously described (10). Metagenomic libraries were sequenced on the Illumina HiSeq 2500 platform, targeting ~2.5 Gb of sequence per sample with 101-bp paired-end reads. Reads were quality controlled by trimming low-quality bases and removing reads of <60 nucleotides. Reads aligning with the human genome were identified with bowtie (40) and filtered out. Samples were profiled taxonomically with MetaPhlAn 2.0 (41) (http://huttenhower.sph.harvard.edu/MetaPhlAn2) and profiled functionally with HUMAnN2 (42) (http://huttenhower.sph.harvard.edu/HUMAnN2). HUMAnN2 maps metagenomic reads to UniRef50 (41) gene families of species identified in the MetaPhlAn2 taxonomic profiling step. Protein-coding sequences in these pangenomes have been preannotated to their respective UniRef50 families, which serve as a comprehensive, nonredundant protein sequence database. Reads that do not align with a known pangenome are separately mapped to the entirety of UniRef50 by translated search with DIAMOND (42). All hits are weighted on the basis of alignment quality and sequence length, with per-species and unclassified hits combined to produce community totals for each protein family (in addition to species-stratified totals) in numbers of reads per kilobase (RPK). RPK units were further normalized to numbers of RPK per million sample reads to account for variation in sequence depth across samples. Principal-component analysis plots were generated with the scikit-learn python package by using species abundance from MetaPhlAn2. Data are available at https://figshare.com/s/5e56cc1a347ef4f1de49 and https://www.ncbi.nlm.nih.gov/bioproject/PRJNA414479.

### GO functional annotation.

We used HUMAnN2 to map UniRef50 gene families to GO terms, which were then aggregated into larger metabolic clusters with the CateGOrizer tool (43). This procedure yielded a comprehensive but manageable set of 13 nonredundant GO biological process terms for comparison of HMP1 and new samples (https://figshare.com/s/5e56cc1a347ef4f1de49).

### Isolation of lipid A for MS analysis.

For analysis of crude biomass, pellets from 10-ml overnight broth bacterial cultures were washed three times in water, methanol, and chloroform (0.8:1:2). Alternatively, purified LPS (200 µg) was used directly. The material was subjected to mild acid hydrolysis at 100°C for 30 min in 12.5 mM sodium acetate buffer, pH 4.5, in the presence of 1% SDS to break the 3-deoxy-d-manno-octulosonic acid linkage, and free lipid A was recovered by two-phase Bligh-Dyer extraction. The lipid A species were analyzed with a MALDI-TOF mass spectrometer (Bruker Ultraflex) equipped with a smartbeam laser at a 2-kHz firing rate. Spectra were acquired in negative-ion linear mode. The matrix used was a saturated solution of 6-aza-2-thiothymine in 50% acetonitrile and 10% tribasic ammonium citrate (9:1, vol/vol). Samples were dissolved in chloroform-methanol (4:1, vol/vol) and deposited on the sample plate, followed by an equal portion of matrix solution (0.5 µl).

### HEK-293 NF-κB reporter cell assays.

HEK-293 cells (5 × 10^4^) stably expressing the NF-κB-inducible Lucia luciferase reporter gene and the genes for either hTLR4, CD14, and MD2 (TLR4-HEK) or hTLR2 and CD14 (TLR2-HEK) (5 × 10^4^) were seeded into the wells of 96-well plates and stimulated with doses of LPS purified from the strains indicated at doses ranging from 10^−3^ to 10^4^ ng/ml for 6 to 8 h. For inhibition assays, cells were stimulated simultaneously with 1 ng/ml LPS purified from *E. coli*. Luciferase activity was measured by BrightGlo (Promega) in accordance with the manufacturer’s instructions. HEK-293 reporter cells were purchased from InvivoGen. All cell lines were tested for mycoplasma contamination with a PCR-based assay by an independent service provider.

### Correlation of function and composition.

Human PBMCs were stimulated in the presence of increasing amounts of fecal LPS or cotreated with 1 ng/ml *E. coli* LPS and increasing doses of fecal LPS from the donors indicated. IL-6, TNF-α, and IL-1β concentrations in supernatants were measured after 20 h of culture and compared to those obtained with LPS treatment alone (0:1). Cytokine concentration upon stimulation at 1 or 10 μg/ml was obtained for LPS from individual stool samples and normalized. Inhibition potency at a ratio of 10:1 or 100:1 was obtained for individual stool samples and normalized. Five independent experiments were performed. Spearman rank correlation between the abundance of individual species and either stimulation or inhibition of IL-6, TNF-α, or IL-1β production was calculated by using pooled data from all experiments. Species that significantly correlate with function for all three cytokines measured after Benjamini-Hochberg correction are shown. Data are available at https://figshare.com/s/5e56cc1a347ef4f1de49.
