# Supplementary figures and images for: Total Lipopolysaccharide from the Human Gut Microbiome Silences Toll-Like Receptor Signaling
Source: mSystems. 2017 Nov 14;2(6):e00046-17. doi: 10.1128/mSystems.00046-17 (PMC5686520; doi:10.1128/mSystems.00046-17)

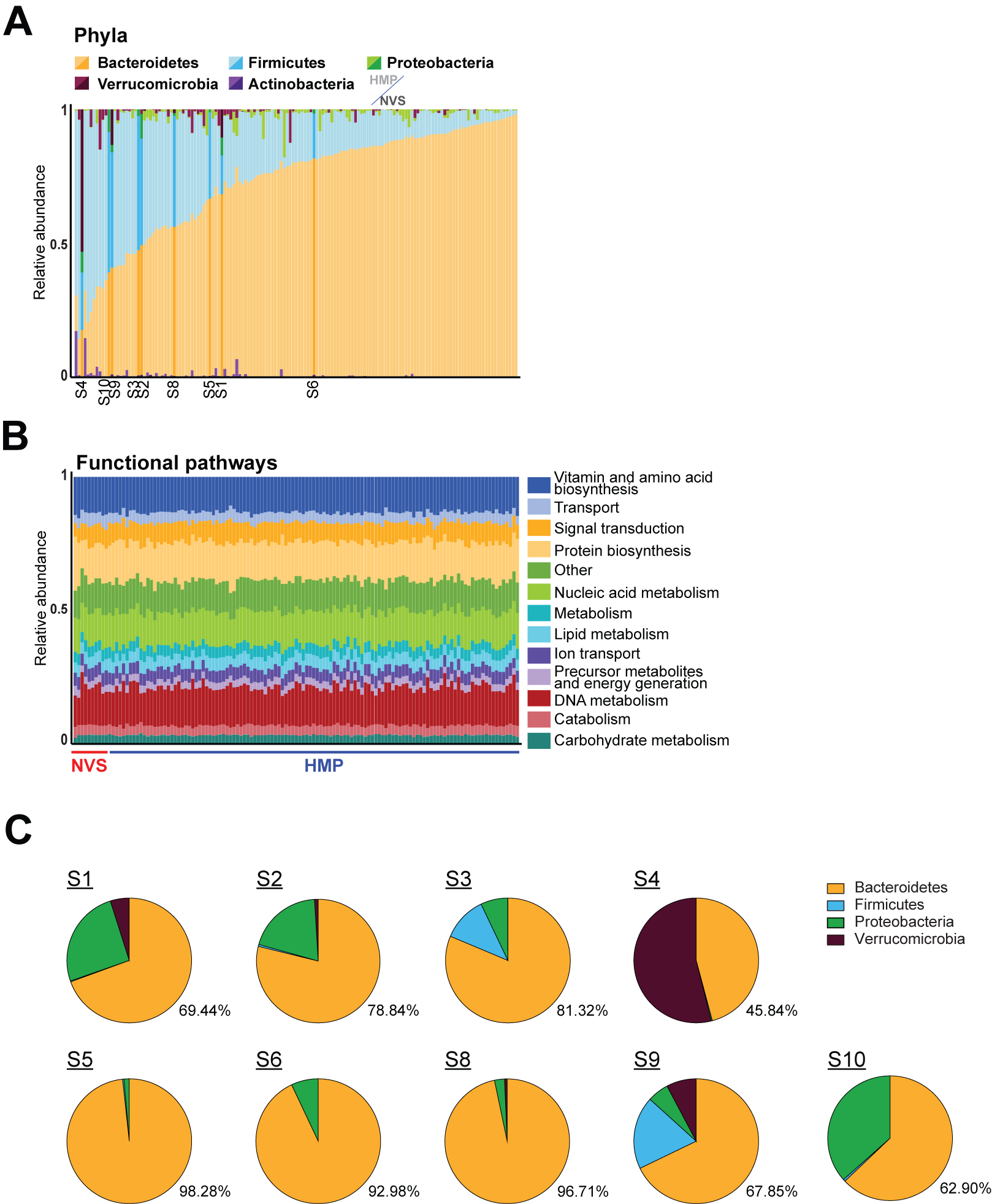

Supplement: FIG S1 [file sys006172148sf1.tif]

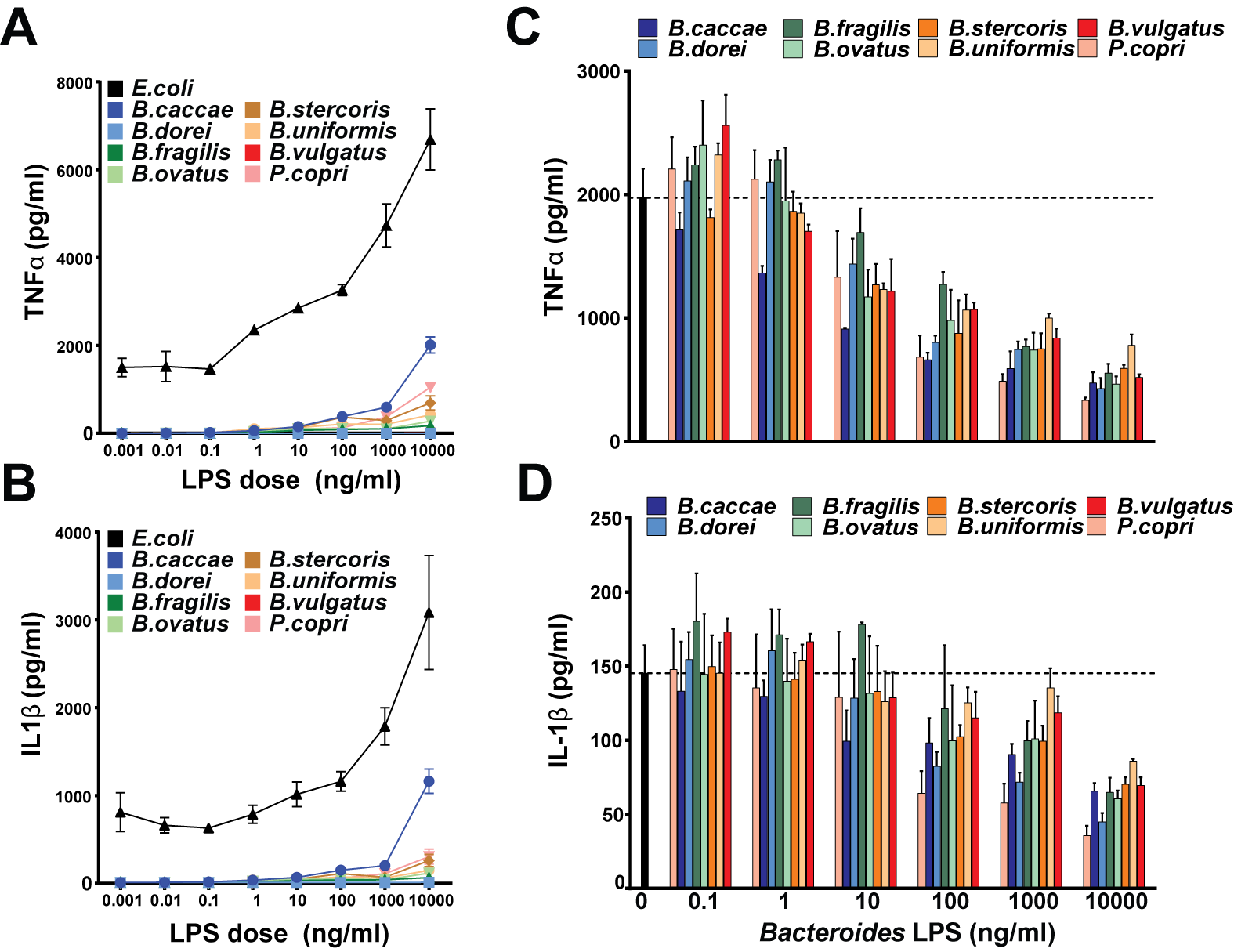

Supplement: FIG S2 [file sys006172148sf2.tif]

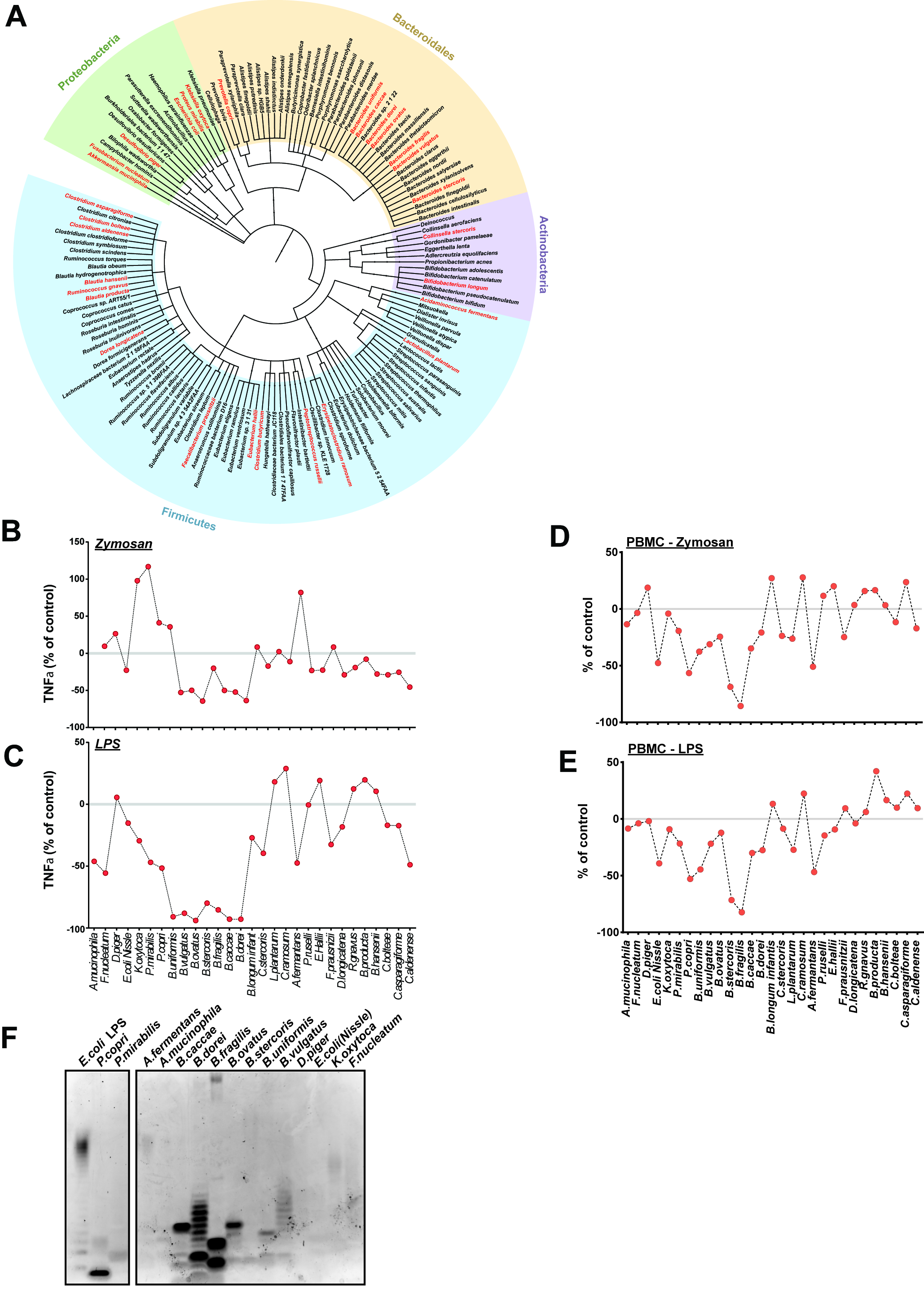

Supplement: FIG S3 [file sys006172148sf3.tif]
